# 280. Monoclonal Antibodies Against SARS-CoV-2 in Fragile Outpatients: Clinical and Laboratory Risk Factors and Protective Role of Vaccination

**DOI:** 10.1093/ofid/ofac492.358

**Published:** 2022-12-15

**Authors:** Rosario Alessandro Cavasio, Neva Braccialarghe, Drieda Zaçe, Ilaria Spalliera, Luigi Coppola, Laura Campogiani, Lorenzo Piermatteo, Maria Concetta Bellocchi, Francesca Ceccherini Silberstein, Iannetta Marco, Loredana Sarmati, Massimo Andreoni

**Affiliations:** Univeristy of Rome Tor Vergata, Rome, Lazio, Italy; University of Rome Tor Vergata, Rome, Lazio, Italy; University of Rome Tor Vergata, Rome, Lazio, Italy; Tor Vergata University of Rome, Rome, Lazio, Italy; Tor Vergata Hospital, Rome, Lazio, Italy; Tor Vergata University of Rome, Rome, Lazio, Italy; University of Rome Tor Vergata, Rome, Lazio, Italy; University of Rome Toer Vergata, Rome, Lazio, Italy; Tor Vergata University, Rome, Rome, Lazio, Italy; University of Rome Tor Vergata, Rome, Lazio, Italy; Tor Vergata University of Rome, Rome, Lazio, Italy; Tor Vergata University of Rome, Rome, Lazio, Italy

## Abstract

**Background:**

To cope with the SARS-CoV-2 pandemic, several treatments were studied and out of these, monoclonal antibodies (MAbs) have shown efficacy to prevent the development of pneumonia after the infection

**Methods:**

We conducted a retrospective, single-center study including patients with SARS-CoV-2 infection, treated with MAbs (bamlanivimab/etesevimab (B/E), casirivimab/imdevimab (C/I) or sotrovimab (S)) from March 2021 to February 2022

**Results:**

We included 504 patients with a median age of 62 years (IQR 49-72), 51% were males and 66% had completed the vaccination schedule according to the current Italian regulations. The most frequent eligibility criteria are summarized in figure 1. As for MAbs combination, patients were treated with B/E (54%), followed by C/I (30%) and S (16%). Outcomes are shown in Table 1.

Nasopharyngeal swab (NPS) negativization time had a positive correlation with patients’ age (r=0.16; p=0.001), C-reactive protein (CRP) (r=0.26; p< 0.001) and creatinine values (r=0.22; p< 0.001) assessed at baseline (infusion day). Time to NPS negativization was 6.9 (95% C.I. [4.5-9.2]) days shorter for vaccinated compared to unvaccinated patients (p< 0.001).

Patients treated with C/I had a negative NPS on average 4.5 (95% C.I.= [1.8-7.3] days earlier than patients treated with B/E; patients who received S reached negativization 6.0 (95% C.I.= [2.2, 9.9]) days earlier than those treated with B/E (p=0.004). Patients with positive outcome had a negative NPS on average 14.3 (95% C.I.= [6.8, 23.1)], 25.5 (95% C.I.= [18.9, 33.4] and 68.3 (95% C.I.= [47.7, 90.2]) days earlier than patients who needed hospitalization and patients who died (p< 0.001, p< 0.001, respectively).

Unvaccinated patients had a higher rate of oxygen support need compared to vaccinated ones (p=0.006). Patients with worse outcomes were significantly older and had higher values of CRP and creatinine at baseline (p=0.04, p< 0.001, p< 0.001, respectively)

Percentage of eligibility criteria of our patients

**Figure:**
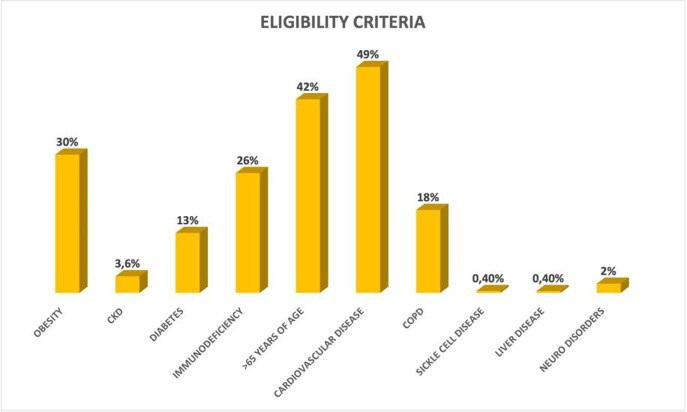

The majority of patients had more than one criterion

Clinical outcome of our patients

**Figure:**
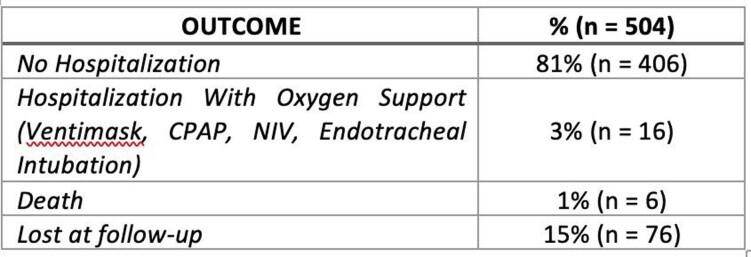

Nasopharyngeal swab was repeated weekly, until negative

**Conclusion:**

MAbs reduce the risk of hospitalization in fragile patients. Vaccinated patients had shorter time of NPS negativization and lower probability of hospitalization. Older age, higher CRP and creatinine values assessed at baseline, correlated with worse outcomes. S was the most effective treatment amongst MAbs used in our study

**Disclosures:**

**All Authors**: No reported disclosures.

